# The Formation of a Highly Oriented Structure and Improvement of Properties in PP/PA6 Polymer Blends during Extrusion-Stretching

**DOI:** 10.3390/polym12040878

**Published:** 2020-04-10

**Authors:** Yu Wang, Wenjie Sun, Song Liu, Huajian Ji, Xin Chen, Huihao Zhu, Haili Zhao, Yulu Ma, Linsheng Xie

**Affiliations:** School of Mechanical and Power Engineering, East China University of Science and Technology, Shanghai 200237, China; apirl.1992@outlook.com (Y.W.); 18818278176@163.com (W.S.); lsezhou@163.com (S.L.); shxiaoji@163.com (H.J.); chenxin198905@aliyun.com (X.C.); zhhxhhy1994@126.com (H.Z.); zhl419wsm@163.com (H.Z.); myl@ecust.edu.cn (Y.M.)

**Keywords:** microfibrilar composites (MFCs), extrusion-stretching, phase morphology, crystalline structure

## Abstract

During the “slit die extrusion-hot stretching” process, highly oriented polyamide 6 (PA6) dispersed phase was produced and retained in the polypropylene (PP) matrix directly. By adjusting the stretching forces, the PA6 spherical phase evolved into the ellipsoid, rod-like microfibril with a decreasing average diameter; then, the PA6 microfibrils broke. Moreover, the effects of the PA6 phases formed in the process of the microfibrillation on PP’s crystallization behaviors were studied systematically. As the stretching forces increased, the crystallization ability and orientation degree of PP crystals improved significantly. Differential scanning calorimetry and polarizing optical microscopy confirmed the formation of PP spherulite, fan-shaped lamellae and a transcrystalline layer under the induction of the PA6 phases with different morphology. In the PP/PA6 microfibrilar composites (MFCs), PP crystals showed smaller average size, more crystals and stronger interface adhesion due to more excellent heterogeneous nucleation ability of the PA6 microfibrils, which made contributions to the improvement of the melt elasticity responses and oxygen barrier properties of the PP/PA6 polymer blends.

## 1. Introduction

It has been well established that melt blending is a cost-effective and practical method for developing new polymer materials [1,2]. The performances of these polymer blends depend not only on the characteristics of each component but also on the phase morphology [3]. Hence, the dispersed phase of the polymer blends is usually processed into a hierarchical structure, such as as fiber, ribbon or lamella, in order to meet specific product demands [4,5,6,7]. 

Fiber-reinforced composites have been widely used in the industry for many years due to their excellent performances [5,8]. However, these fibers, such as carbon fiber and glass fiber, could cause abrasive wear of the processing equipment. Additionally, some fibers could be broken down into fragments during processing, thereby reducing the performances of the composites [9,10]. Recently, the microfibrillated cellulose fibers have been used to modify polymer materials [11,12,13,14]. Farris et al. [15] prepared microfibrillated cellulose-modified polypropylene (PP) composites, the elastic modulus of which increased by approximately 55%. Lepetit et al. [13] improved the interface adhesion between the low-density polyethylene (LDPE) and the microfibrillated cellulose by the allylation and propargylation of the microfibrillated cellulose fibers. Nevertheless, the poor dispersion and intrinsic rigidity of the cellulose fibers still have a significantly negative influence on the modification effects. In recent years, much attention has been paid to the preparation of the microfibrilar composites (MFCs), wherein other polymer fibrils are in situ generated in a thermoplastic polymer matrix [16,17,18]. This is considered an economic and high-throughput method for novel polymer blends or composites’ preparation, and different from the conventional melt-blending of the polymer and the fiber fillers.

In general, the stretch or shear flow is applied on the blends for the sake of the effective fibrillation of the polymer blends. It has been verified that the spherical dispersed domains went through deformation and coalescence, and then evolved into long and even endless fibrils during flow field [18,19]. Furthermore, many factors—the draw ratio [20,21], viscosity ratio [21,22], components ratio [3,23], interfacial tension [24], etc.—have been studied for the sake of gaining control of the morphological development of the dispersed phase in the matrix. For example, Jiang et al. [23] prepared high-density polyethylene (HDPE)/polystyrene (PS) MFCs by the vibration injection molding (VIM). They found that an increase of the PS content caused phase morphology and size changes. Wang et al. [21] also reported that a lower viscosity ratio of the dispersed phase to the matrix facilitated the deformation and coalescence of the dispersed phase in order to form highly oriented microfibrils. Furthermore, the phase morphology with the microfibrilar shape has shown great potential in improving the crystalline behavior of the matrix [25,26]. Specially, the microfibrils could induce the formation of the additional oriented crystals, which increases intermolecular interaction and block chain segment movement, in comparison with the spherulites and loose lamellae [27,28], and afterwards strongly influences the final performances of the polymer blends.

The MFCs are currently prepared in three steps, including: (i) melt blending of the dispersed phase and the matrix at a temperature above the melting points of the two phases; (ii) hot or cold stretching of the extrudates to achieve the fibrillation process; (iii) injection or compression molding at the temperature between the melting temperatures of the two phases (isotropization) [29,30,31]. The isotropization process produces the isotropic matrix and the randomly oriented microfibrils, and it has been verified to have an effect on the phase morphology, crystal structure and performance of a polymer blend. For example, Xia et al. [32] prepared the HDPE/polycarbonate (PC) MFCs by the hot-stretching and gas-assisted injection molding (GAIM) technique. The classic shish-kebabs, with typical transcrystallinity and hybrid shish-kebab superstructures, were observed in this study. The results showed that the yield strength and tensile modulus of the PP/PC MFCs improved by 66% and 17%, respectively, compared to that of the HDPE/PC blends molded by the common injection molding (CIM). Zhao et al. [33] reported that the increased mold temperature could induce the formation of the lamella, fan-shaped *β*-crystals and transcrystals around the in situ poly(ethylene terephthalate) (PET) microfibrils. However, there are also some studies about the negative effects of the injection molding process. For example, the injection molding process caused a reduction in the microfibrilar aspect ratio [20,33,34], which was a key to determining the performances of the MFCs. Moreover, little attention extensively focused on the effects of the dispersed phases formed in the process of the microfibrillation on the crystal structure and performance of the matrix in the polymer blends.

In this work, we chose PP as the matrix and polyamide 6 (PA6) as the dispersed phase, respectively, due to their processabilities and reasonable prices. The casting equipment (“slit die extrusion-hot stretching”) was used to in situ generate the highly oriented structure in the PP/PA6 polymer blends. By adjusting the stretching forces, the PA6 dispersed phase was stretched into a different morphology. The effects of the PA6 dispersed phases on the crystallization behaviors and crystal structure of the PP matrix were investigated systematically to illuminate the formation mechanism of the highly oriented structure. Furthermore, under the synergistic effects of the PA6 microfibrils and PP crystals, the PP/PA6 MFCs showed more excellent melt elasticity responses and oxygen barrier properties.

## 2. Materials and Methods

### 2.1. Materials and Sample Preparation

The PP with a melt flow rate of 2.5 g/10 min (230 °C, 2.16 kg, ASTM Standard D1238) was provided by Lyondellbasell Industries Corporation Ltd., Hoofddorp, Netherlands (tradename PF814). The PA6 with a melt flow rate of 130 cm^3^/10 min (275 °C, 5.0 kg, ISO 1133) was provided by BASF SE Corporation Ltd., Shanghai, China (tradename B27E). It has a density of 1.14 g/cm^3^ (ISO 1183) and a melting temperature of 220 °C.

After drying for 24 h at 80 °C, the PP and PA6 (85/15 mass fraction) were compounded and extruded into the pellets in a twin-screw continuous extruder (ECM30, homemade) with a diameter of 30 mm and a length-to-diameter ratio of 40. The barrel temperatures of the extruder were set at 50, 100, 150, 180, 200, 210, 220, 230 and 220 °C from the feed section to the die. The screw speed was set to 500 rpm.

After drying for 12 h at 80 °C, the PP/PA6 compounded pellets were used for the film extrusion in a single-screw extruder (FDHU-20, D = 20 mm, L/D = 28, POTOP, Guangzhou, China). The barrel temperature was set at 230 °C and the screw speed was 40 rpm. The extrudates were stretched from the slit die (0.5 mm of thickness and 100 mm of width) by the rollers. Different stretch forces were obtained by adjusting the velocity of the rollers to 0, 0.5, 1.5, 2.5 and 3.5 m/min. According to the definition of the stretch ratio (*λ*), that is, the ratio of area of the transverse section of unstretched extrudate to that of stretched extrudate, the corresponding stretch ratios were 1, 2, 7, 11 and 16, respectively. In this paper, the stretch ratio of 1 was defined as no stretch for the extrudates, the stretch ratio of 2–7 was defined as relatively low stretch forces for the extrudates and the stretch ratio of 11–16 was defined as relatively high stretch forces for the extrudates, respectively. Finally, the extrudates were directly shaped and collected by the take-up rollers. For comparison, pure PP were also prepared with the same thermal history.

### 2.2. Characterizations

*Scanning electron microscopy (SEM)*: the samples were first placed in liquid nitrogen for 30 min and then cryogenically fractured along the stretching direction. Some of the cryo-fractured surfaces were used directly for the SEM observation. For others, they were immersed in xylene for 1.5 h at 140 °C, and after the time-equlibrium state was reached, PP phases were extracted. Then, all treated samples were ultrasonically cleaned in the distilled water. After being dried and covered with a thin layer of gold, these samples were observed by an SEM (JSM-6360LV, Hitachi, Tokyo, Japan) device. Image-Pro Plus 6.0 was used to characterize the diameter of the PA6 dispersed phase morphology.

*Differential scanning calorimetry (DSC)*: A DSC (TA-Q100, TA Instruments, New Castle, PA, USA) device was employed to investigate the crystallization behaviors of the samples in a nitrogen atmosphere.

During the non-isothermal crystallization, the samples (approximately 5 mg) were heated to 200 °C and treated isothermally at 200 °C for 10 min to eliminate thermal history. Then, the samples were cooled to 50 °C at a cooling rate of 10 °C/min for non-isothermal crystallization of the PP phases. Next, the samples were heated to 200 °C at a heating rate of 10 °C/min to determine the melting temperature and heat flux of the PP phases.

During the isothermal crystallization, the samples (approximately 5 mg) were rapidly cooled from 200 to 125 °C at a cooling rate of 40 °C/min after eliminating thermal history. Then the testing temperature was kept at 125 °C until the full crystallization of the PP matrix occurred. The relative crystallinity as a function of time can be expressed by Equation (1):

(1) $$X\left(t\right)=\frac{\int_{0}^{t}\left(\frac{dH}{dt}\right)dt}{\int_{0}^{\infty }\left(\frac{dH}{dt}\right)dt}$$

where *t* = 0 is the instant when thermal equilibrium is obtained, and *t* = ∞ is the instant when the crystallization is completed. $\int_{0}^{t}\left(\frac{dH}{dt}\right)dt$ is the heat generated from the attainment of thermal equilibrium to time *t*.

*Wide-angle X-ray scattering (WAXS)*: A rotating anode x-ray powder diffractometer (18KW/D/max2550VB/PC, Rigaku Corporation Ltd., Tokyo, Japan) was used to characterize the WAXS patterns of the samples. The tests were performed under ambient conditions, using a Ni filtered Cu−Kα radiation with a wavelength (*λ*) of 1.5406 Å. The generator was with a voltage of 40 kV and a current of 100 mA. The scan angle was in the range of 3° – 50°at a speed of 0.02 (2*θ*/s).

*Two-dimensional small-angle X-ray scattering (2D-SAXS)*: A SAXS device (beamline BL16B1, Shanghai Synchrotron Radiation Facility, SSRF, Shanghai, China) was used to study the crystal lamellar structure of the samples at room temperature. A Pilatus 300 K detector (487 × 619 pixels with a pixel size of 172 mm) was performed to collect the SAXS patterns. The X-ray wavelength was 0.124 nm, and the sample-to-detector distance was set at 4375 mm. In 2D-SAXS patterns, the scattering vectors along and perpendicular to the stretching direction were defined as *q*_1_ and *q*_2_, respectively. The intensity distribution curve was obtained by integrating the intensity at each *q*_1_ for a certain range of *q*_2_, as shown in Equation (2):

(2) $$I(q_{1})=\int_{q_{2,a}}^{q_{2,b}}I(q_{1},q_{2})dq_{2}$$

where *q_2,a_* and *q*_2,b_ are selected in such a way that the scattering peak covers the range of the integration. The long period (*L*) of the samples could be calculated by the Bragg equation (Equation (3)):

(3) L=2π/q*

where q* is the corresponding value of the peak position in the intensity distribution curves.

*Polarizing optical microscopy (POM)*: A POM (Eclipse Ci POL, Nikon, Tokyo, Japan) and a heating stage (HCS302, Instec, Naperville, IL, USA) were used to observe the crystal structures of the samples. Thin slices were cut from the samples by a microtome, and they were used for POM observation better. During testing, a thin slice of each sample was heated to 200 °C on the heating stage, cooled rapidly to 125 °C and kept at 125 °C until full crystallization occurred. The crystallization processing of the samples was observed and recorded by POM.

*Dynamic rheology testing*: A parallel-plate rheometer (Bohlin Gemini 2, Malvern Instruments Inc., Malverne, UK) was used to measure the rheological property of the samples. The diameter of the plate was 25 mm, and the gap between plates was 1 mm. The testing was performed at 200 °C, over a range of frequencies from 7.96 × 10^−3^ Hz to 7.96 × 10^1^ Hz. The strain was controlled at 5.0%.

*Oxygen barrier property testing*: A film permeability testing device (VAC-V2, Labthink, Jinan, China) was used to characterize the oxygen barrier properties of the samples at room temperature with 50% relative humidity. The film sample was placed in the middle of two chambers. An O-ring and a silicone grease were placed between the film and the chamber walls to prevent gas leakage. The volume of oxygen that permeated from the film sample was measured by in situ sensors connected to a data recording computer. The gas permeability *P* could be calculated using the oxygen transmission rate (OTR) through the film sample by means of Equation (4):

(4) $$P=\frac{d\Delta V_{gas}}{A\Delta p\Delta t}$$

where *d* (cm) is the film thickness, Δ*V*_gas_ (cm^3^) is the change of oxygen volume permeated from the film samples, *A* (cm^2^) is the film area, Δ*p* (Pa) is the partial pressure difference across the film samples and Δ*t* (s) is the duration of the testing.

## 3. Results and Discussion

### 3.1. Phase Morphology Development

**Figure 1:**
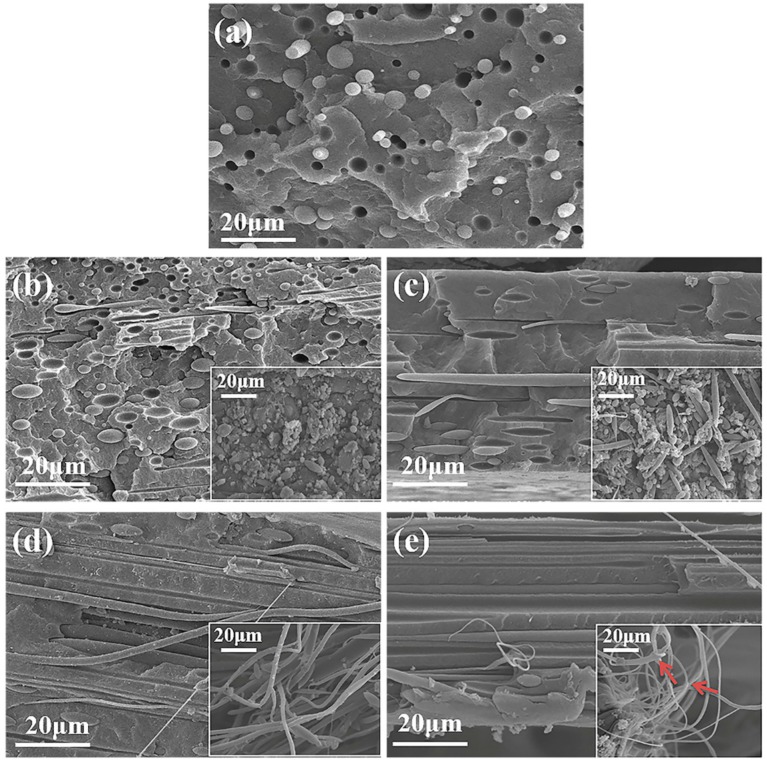
Scanning electron microscopy (SEM) images of cryo-fractured surfaces of the polypropylene (PP)/polyamide 6 (PA6) blends: (**a**–**e**) the samples with stretch ratios of 1, 2, 7, 11 and 16, respectively. The inset images are SEM images of cryo-fractured surfaces of the PP/PA6 blends after etching PP matrix.

The morphologies of the cryo-fractured surfaces of the PP/PA6 blends with different stretch ratios are shown in Figure 1. After etching the PP matrix, the exposed PA6 phases were clearly observed in the corresponding inset images. In the case of the PP/PA6 blends, the typical sea-island phase morphology was observed in Figure 1a. It could be seen that the spherical PA6 domains were immersed in the PP matrix, and there were some gaps and holes on the cryo-fractured surfaces, indicating poor thermodynamic compatibility between the PP and PA6 domains. After stretching, the PA6 dispersed phases began to orient and deform. When the stretch ratio was relatively low, the PA6 phases showed slight deformation and evolved into ellipsoid (*λ* = 2) and rod-like (*λ* = 7), as shown in Figure 1b,c. The transition of the spherical PA6 phases to microfibrils occurred at the stretch ratio of 11. Well-aligned PA6 microfibrils were clearly observed in Figure 1d, with a high aspect ratio and orientation degree along the stretching direction. When the stretch ratio increased to 16, finer PA6 microfibrils were observed in Figure 1e. However, at this time, there were some microfibril breaks observed, as indicated by the arrows. This results in the stretched molten droplets being unable to withstand the large extensional deformation due to growing disturbances at the interface, when the stretch stress is relatively high [20]. The breakage of the microfibrils might lead the aspect ratio of the microfibrils to no longer increase. Hence, the degree of the in situ fibrillation of the PP/PA6 blends increases with increasing stretch ratio until the PA6 microfibrils break.

**Figure 2:**
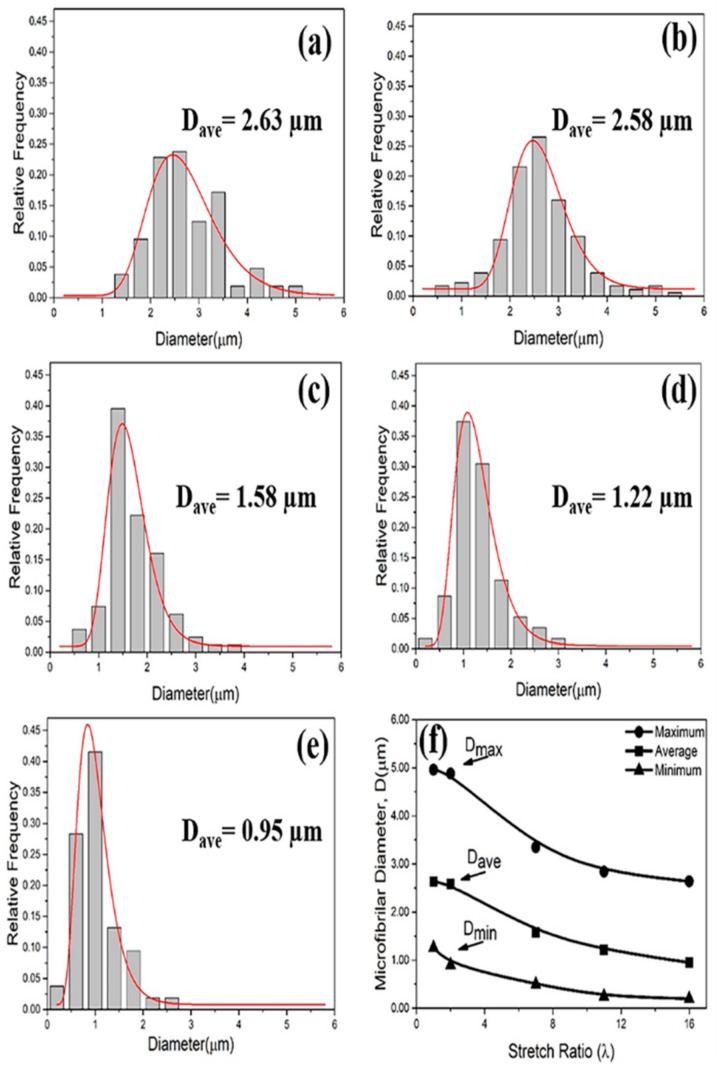
(**a**–**e**) The diameter distribution of the PA6 domains in the PP/PA6 blends with the stretch ratio of 1, 2, 7, 11 and 16. The average diameters (*D*_ave_) are marked. (**f**) The maximum diameter (*D*_max_), average diameter (*D*_ave_) and minimum diameter (*D*_min_) of the PA6 domains as functions of the stretch ratio of the PP/PA6 blends.

The diameter distribution of the PA6 dispersed phases in the PP/PA6 blends with different stretch ratios is shown in Figure 2a–e. The average diameter (*D*_ave_), fitted by log-normal distribution function, is also marked. The maximum diameter (*D*_max_), average diameter (*D*_ave_) and minimum diameter (*D*_min_) of the PA6 dispersed phases as a function of the stretch ratio are shown in Figure 2f, where all of them decreased gradually with the increasing stretch ratio. Specifically, when the stretch ratio increased from 1 to 16, the *D*_ave_ of the PA6 dispersed phases decreased from 2.63 to 0.95 μm, and the decreasing trend of the diameters gradually slowed down. Moreover, the PP/PA6 blends with higher stretch ratios showed narrower diameter distributions, as shown in Figure 2a–e. Generally, the capillary number *Ca* (Ca=ηmεR/σ, where *ηmε* represents the hydrodynamic stress and *σ/R* represents the interface stress), as a dimensionless parameter, could determine the deformation of the dispersed phase morphology. The hydrodynamic stress (*ηmε*) is responsible for deforming the droplet into an elongated, fibrillar structure, and the interfacial stress (*σ/R*) is responsible for minimizing the interfacial energy by retaining the spherical morphologies of the dispersed phases [35]. When the value of *Ca* is higher than a critical capillary number (*Cr*), the hydrodynamic stress is sufficient to overcome the interfacial stress; then, the deformation of the dispersed phase morphology occurs. The deformation degree of the dispersed phase morphology could be evaluated by a dimensionless parameter *D* (*D = L-B/L+B*, where *L* and *B* represent the long and short axes of the dispersed phase, respectively). Meanwhile, as the hydrodynamic stress increases constantly, the phase interface disturbance also increases, which eventually results in the deformed dispersed phase breaking up.

### 3.2. Crystallization Behavior

**Figure 3:**
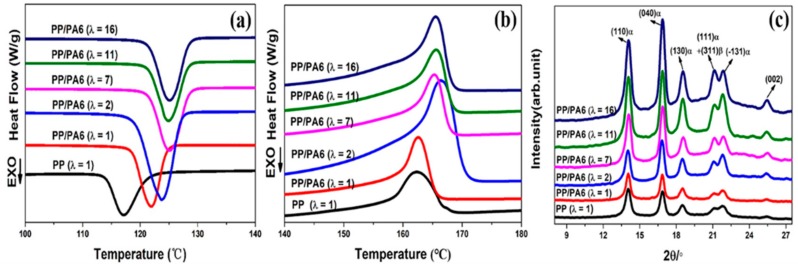
Differential scanning calorimetry (DSC) thermograms of non-isothermal crystallization for the pure PP and the PP/PA6 blends with the stretch ratios of 1, 2, 7, 11 and 16: (**a**) crystallization curves; (**b**) melting curves; (**c**) wide-angle X-ray scattering (WAXS) patterns of the pure PP and the PP/PA6 blends with stretch ratios of 1, 2, 7, 11 and 16.

The DSC crystallization curves for the pure PP and the PP/PA6 blends with different stretch ratios are plotted in Figure 3a. The thermal properties’ parameters, including onset crystallization temperature (*T*_o_) and peak crystallization rate temperature (*T*_p_) are summarized in Table 1. It could be seen that, in comparison with that of the pure PP, the introduction of the PA6 dispersed phases shifted the *T*_o_ and *T*_p_ of the PP matrix to a higher level. This indicates that PA6 dispersed phase has an obvious heterogeneous nucleation effect on the crystallization of the PP matrix. Moreover, as the stretch ratio of the PP/PA6 blends increased, the *T*_o_ and *T*_p_ of the PP matrix improved gradually. They tended to be stable at the stretch ratio of 16, where *T*_o_ and *T*_p_ were 128.4 and 125.3 °C, respectively. This demonstrates that the microfibrilar PA6 phases have more noticeable heterogeneous nucleation effects on the crystallization of PP matrix. This is because, in comparison with the precursor domains (sphere, ellipsoid and rod-like), the larger specific surface areas of the microfibrilar domains could provide more heterogeneous crystal nucleation sites, and then promote the nucleation and growth of the PP matrix.

Figure 3b presents the DSC melting curves for the pure PP and the PP/PA6 blends with different stretch ratios. The melting temperatures (*T*_m_) and degrees of crystallinity (*X*_c_) of the PP matrix in different samples are summarized in Table 1. It could be seen that the *T*_m_ of the PP matrix was higher after the introduction of the PA6 dispersed phases. However, the *T*_m_ and *X*_c_ of the PP matrix in the PP/PA6 blends subjected to stretching almost kept unaltered. This could be ascribed to: (i) the physical hindrance originated from the entangled PA6 microfibrils reduces PP chains’ mobility, and (ii) the physical crosslinking of the PP crystals increases the imperfections of PP crystals [36]. The WAXS patterns for the pure PP and the PP/PA6 blends with different stretch ratios are shown in Figure 3c. The peaks are marked corresponding to those of the PP crystals. Obviously, as the stretch ratio of the PP/PA6 blends increased, the peak intensities of the PP matrix were much more prominent. As is known, the peak intensities of the WAXS patterns are relative to the amount and the orientations of the crystals [37]. In consideration of almost unchanged crystallinity, the great increases in the peaks intensities could be ascribed to a large area of orientation arrangement of the PP crystals in the PP/PA6 MFCs.

In addition, the size changes of the PP crystals in different samples also were analyzed by WAXS patterns. The typical characteristic diffraction peaks of the lattice planes (110) and (040) of the PP *α*-crystals were chosen for investigating the changes of the crystals’ size. The crystal’s size can be calculated according to the Scherrer equation (Equation (5)):

(5) D=Kλ/βcosθ

where *D* is the crystals’ size, *K* is constant, *λ* is the wavelength of the X-ray, *β* is the full width at half maximum from WAXS patterns. When *θ* is fixed, the crystals’ size is inversely proportional to the width of the half peak; namely, the smaller values of 1/*β*cos*θ* represents the smaller crystals’ size. By the calculation and comparison, as the stretch ratio increased, the size of the PP’s crystals had a decreasing tendency. In the PP/PA6 MFCs, the size of the PP’s crystals showed the smallest values. More detailed data were shown in the Tables S1 and S2 of the Supplementary Materials.

**Figure 4:**
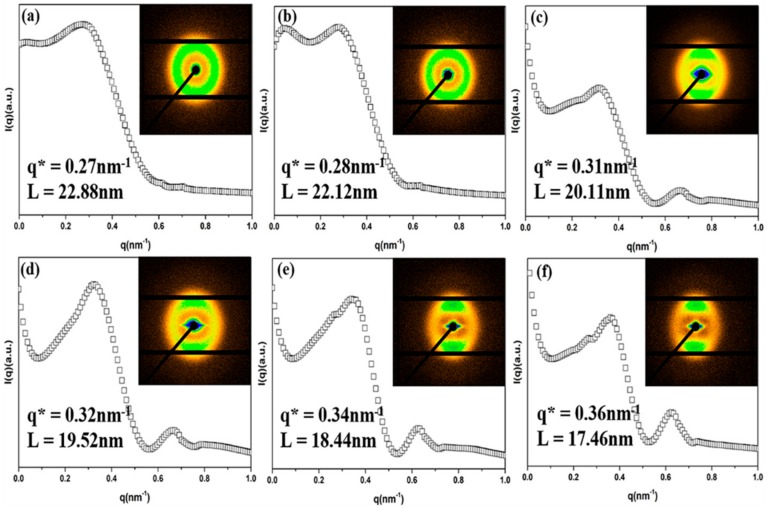
Representative two-dimensional small-angle X-ray scattering (2D-SAXS): patterns and intensity distribution curves in (**a**) pure PP and (**b–f**) PP/PA6 blends with the stretch ratios of 1, 2, 7, 11 and 16, respectively.

The representative 2D-SAXS micrographs of the pure PP and the PP/PA6 blends with different stretch ratios are shown in Figure 4a–f. In comparison with the pure PP (2D-SAXS patterns of Figure 4a), the PA6 domains caused weak circular scattering in the 2D-SAXS patterns of the PP/PA6 blends (Figure 4b). It is worth noticing that, for the PP/PA6 blends subjected to stretching, all showed a pair of symmetrical triangular streaks in the equatorial direction and two arc-like lobes patterns in the meridional direction, as shown in the 2D-SAXS patterns of Figure 4c-f. Additionally, with the increase of the stretch ratio of the PP/PA6 blends, the scattering intensity became stronger, indicating an improved molecular orientation [38]. This is consistent with the WAXS results. The SAXS intensity distribution curves and the calculated long periods of different samples are shown in Figure 4a–f. The peak of the pure PP appeared near q* = 0.27 nm^−1^, and the corresponding long period (*L*) was 22.88 nm. As the stretch ratio of the PP/PA6 blends increased from 1 to 16, the peak (q*) increased from 0.28 to 0.36 nm^−1^, and the corresponding long period (*L*) decreased from 22.12 to 17.46 nm. The reduction of 4.66 nm of *L* could be a result of the formation of more densely packed PP lamellae in the PP/PA6 MFCs [39]. The secondary peak could be observed in the PP/PA6 blends subjected to stretching, as shown in the SAXS intensity distribution curves of Figure 4c–f, and the corresponding long period was around 10 nm. This might be ascribed to the formation of the PP shish-kebab induced by self-nucleated stretched chains. In the process of stretching, in addition to the deformation of phase morphology, some PP chains were stretched from curly state to oriented state. Meanwhile, the flow amplification effects of the deformed PA6 dispersed phases also could result in the formation of more stretched PP chains and hinder their relaxation more effectively [32]. Then, these stretched PP chains could act as row nuclei and capture some random PP chains to form shish-kebab.

**Figure 5:**
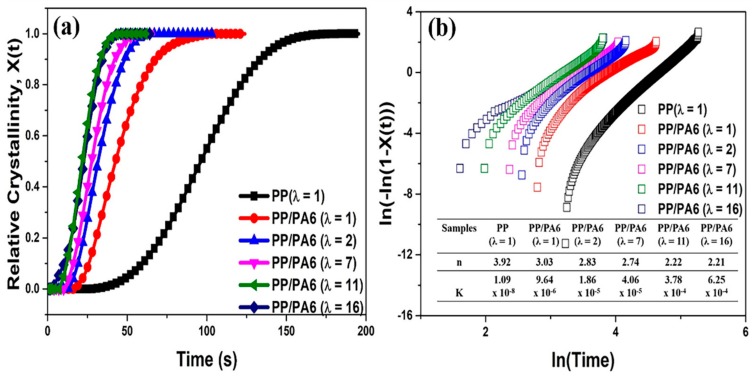
DSC thermograms of the isothermal crystallization for the pure PP and the PP/PA6 blend with the stretch ratios of 1, 2, 7, 11 and 16 at 125 °C: (**a**) Relative crystallinity, *X*(*t*), as a function of the crystallization time (*t*) calculated from DSC; (**b**) isothermal crystallization kinetics analyzed using the Avrami equation. The Avrami parameters, Avrami exponent (*n*) and crystallization rate constant (*K*), are summarized in built-in table of Figure 5b.

The evolution of the relative crystallinity, *X*(*t*), as a function of the crystallization time (*t*) during isothermal crystallization at 125 °C is presented in Figure 5a. Obviously, as the stretch ratio of the PP/PA6 blends increased, the duration for the complete crystallization of the PP matrix shortened. Using the Avrami Equation (6) [40], the isothermal crystallization kinetics were investigated:

(6) ln(−ln(1−X(t)))=lnK+nlnt

where *n* is the Avrami exponent, representing the dimensionality of the crystal growth, and *K* is the overall crystallization rate constant, involving both tge nucleation and growth rate of the crystals. Figure 5b presents isothermal crystallization kinetics of the PP matrix in different samples. The *n* values of the PP crystals in the pure PP and the PP/PA6 blends were close to 4.00 and 3.00, respectively, indicating the formation of the PP spherulite [41]. The difference is that the spherulite in the pure PP is induced by the homogeneous nuclei, and the spherulite in the PP/PA6 blends is induced by the PA6 heterogeneous nuclei. Furthermore, the *n* values of the PP crystals decreased as the stretch ratio of the PP/PA6 blends increased. Under a relatively low stretch ratio (*λ* = 2–7), the *n* value was around 2.80, demonstrating that, in addition to the formation of PP spherulite, there could be some PP lamellae in the PP/PA6 blends. Under a relatively high stretch ratio (*λ* = 11–16), the *n* value was around 2.20, indicating that the PP crystals mainly exist in the form of lamellae in the PP/PA6 MFCs. From the overall crystallization rate constant (*K*), it could also be found that the crystallization rate of the PP matrix was enhanced significantly from 9.64 × 10^−6^ to 6.25 × 10^−4^, when the PA6 dispersed phases were stretched from sphere to microfibrils.

**Figure 6:**
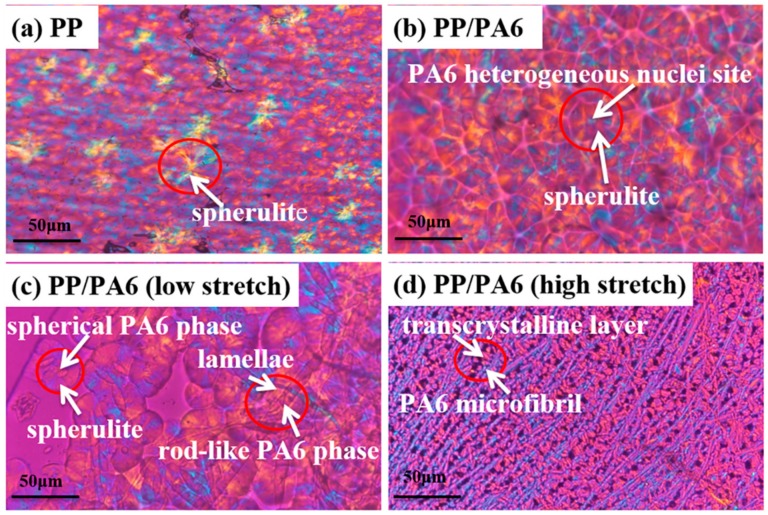
Polarizing optical microscopy (POM) micrographs of (**a**) pure PP, (**b**) PP/PA6 blends, (**c**) PP/PA6 blends with low stretch and (**d**) PP/PA6 blends with high stretch.

To further observe the PP’s crystal structure, the polarizing micrographs of the pure PP and the PP/PA6 blends with different stretch ratios are presented in Figure 6a–d. During the isothermal crystallization at 125 °C, self-nucleating spherulite appeared in the pure PP, as shown in Figure 6a. In the PP/PA6 blends, PP chains grew into spherulite induced by the PA6 heterogeneous nuclei, as shown in Figure 6b. These spherulite showed blurred crystal boundaries due to rapid heterogeneous nucleation effects of the PA6 dispersed phases. In the PP/PA6 blends with a relatively low stretch ratio (*λ* = 7), PP spherulite and fan-shaped lamellae were observed simultaneously in Figure 6c. The growth of the two different PP crystal structure was induced, respectively, by the PA6 spherical phases and rod-like phases. This is well consistent with the results of the isothermal crystallization kinetics. Figure 6d shows the POM micrographs of the PP/PA6 MFCs. It could be seen that the oriented PP lamellae wrapped slightly and perpendicularly the surfaces of the PA6 microfibrils, which presented the typical features of the transcrystalline layers. The formation of the transcrystalline layers implies that the interface adhesion between the PP matrix and the PA6 dispersed phase are improved [27]. Moreover, compared with those of Figure 6a–c, the PP crystals in the PP/PA6 MFCs showed smaller average sizes and were present in a greater quantity. This result is in good agreement with the crystal size changes calculated from the WAXS curves. Based on a higher heterogeneous nucleation ability of the PA6 microfibrils, this could be a result of the constraints of densely adjacent nuclei and geometric space [36]. In addition, a higher specific surface area of these small PP crystals could hinder PP chains’ mobility in the amorphous domains. This will have a significant effect on the properties of the PP/PA6 blends.

**Figure 7:**
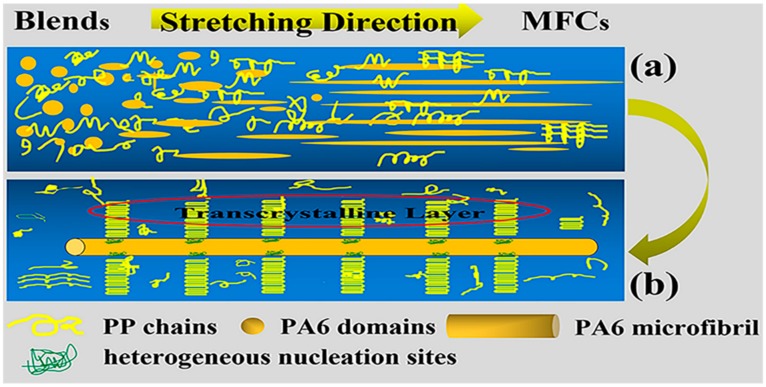
A schematic diagram for (**a**) the evolution of the phase morphology and (**b**) the internal structure of the PP/PA6 microfibrilar composites (MFCs).

Based on above observation and analysis, the diagrams for the phase morphology evolution and internal structure of the PP/PA6 MFCs are presented in Figure 7a,b, respectively. As shown in Figure 7a, the PA6 dispersed phase in the PP/PA6 blends deformed from sphere, ellipsoid and rod-like into microfibrils in the process of the in situ microfibrillation. This is a synergistic result of the deformation and coalescence of the droplets during stretching flow. The attempt at describing the internal structures of the PP/PA6 MFCs is shown in Figure 7b. The long axis of the PA6 microfibrils was parallel to the stretch direction, and well-aligned PA6 microfibrils provided abundant heterogeneous nuclei, which could absorb initial PP molecular chains to locate on their surfaces effectively. Then these PP chains folded into the oriented lamellae and grew epitaxially into transcrystalline layers due to the constraints of densely adjacent nuclei and growth space. The formation of the transcrystalline layers could enhance the interface adhesion between the PP matrix and the PA6 dispersed phase, and then have a significant effect on the properties of the PP/PA6 polymer blends.

### 3.3. Rheological and Oxygen Barrier Properties

**Figure 8:**
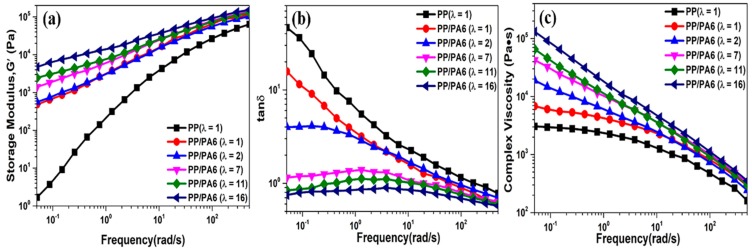
Shear rheological behavior of the pure PP and the PP/PA6 blends with different stretch ratios at 200 °C. (**a**–**c**) The dependencies of storage modulus, loss tangent and complex viscosity on the frequencies, respectively.

The shear rheological responses of the pure PP and the PP/PA6 blends with different stretch ratios are presented in Figure 8. The testing temperature was set at 200 °C, which was above the melting temperature of the PP matrix and low enough to prevent the PA6 dispersed phases from melting. The dependencies of the storage modulus (*G*’) and the loss tangent (tan *δ*, the ratio of loss modulus to storage modulus) on the frequencies are shown in Figure 8a,b, respectively, which reflects the elasticity of the PP melt. Beyond the frequencies, there was a classic viscoelastic behavior for the pure PP, where the *G*’ and the tan *δ* showed an obvious dependence to the frequency. The increase of the stretch ratio of the PP/PA6 blends caused an increase in the *G*’ and a decrease in the tan *δ* of the PP melt at low frequencies. Especially at the stretch ratio of 16, the *G*’ was improved by an impressive 3.5 orders of magnitude, and the value of the tan *δ* was nearly 1. At high frequencies, the *G*’ and tan *δ* of the PP melt showed stable values. These phenomena are indicative of a variation in the viscoelastic responses of the PP melt from “liquid-like” to “gel-like”; namely, an increase in the elasticity [42]. The increase of the PP melt’s elasticity could be ascribed to a reduction of the PP chains’ mobility in the amorphous domains. The introduction of the PA6 dispersed phase could reduce the PP chains’ mobility. Additionally, this effect is more significant in the PP/PA6 MFCs due to a larger specific surface area of the PA6 microfibrils [42]. Moreover, a higher heterogeneous nucleation ability of the PA6 microfibrils could promote the formation of more, smaller PP crystals. Hence, the physical crosslinking effect between the PP crystals is enhanced, which also could reduce the PP chains’ mobility. In addition, the formation of a physically entangled network, as a result of the topological interactions of the PA6 microfibrils, is another decisive factor for the increase of the elasticity. This entangled network structure could store deformation energy and reduce free volume more effectively, and then improve the elastic responses [25]. The dependence of the complex viscosity on the frequencies is also shown in Figure 8c. The complex viscosity of the pure PP exhibited typical shear thinning behaviors. As the stretch ratio increased, the complex viscosity of the PP/PA6 blends enhanced significantly. This is ascribed to the fact that the entangled PA6 microfibrils restricted the long-range motion of the PP chains and prevented them from complete relaxation when subjected to an external force.

**Figure 9:**
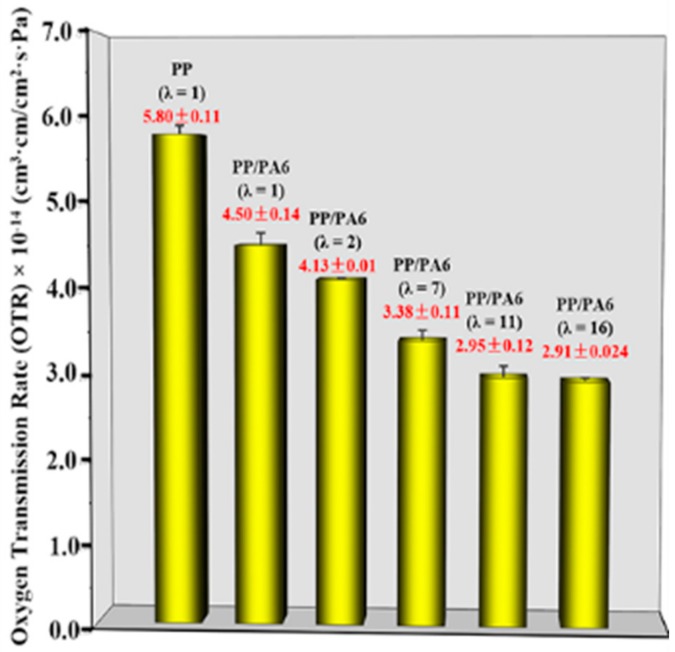
The oxygen transmission rates of the pure PP and the PP/PA6 blends with different stretch ratios.

The oxygen transmission permeabilities (OTR) of the pure PP and the PP/PA6 blends with different stretch ratios are plotted in Figure 9. In comparison with the OTR of the pure PP (5.8 × 10^−14^ cm^3^·cm·cm^−2^·s^−1^·Pa^−1^), the additions of the spherical PA6 phases decreased the OTR to 4.50 × 10^−14^ cm^3^·cm·cm^−2^·s^−1^·Pa^−1^. This is a result of the PA6 dispersed phase as a barrier filler. As the stretch ratio increased, the OTR of the PP/PA6 blends decreased gradually; in other words, there was an improvement in the oxygen barrier properties. Specifically, at the stretch ratio of 16, the OTR of the PP/PA6 MFCs was as low as 2.91 × 10^−14^ cm^3^·cm·cm^−2^·s^−1^·Pa^−1^. This is caused by a synergistic effect of the PP crystals’ changes and PA6 phase morphology deformation. For one, the PA6 microfibrils as the excellent heterogeneous nucleation agents promote the formation of numerous small PP crystals. These crystals improve the barrier properties due to their impermeable nature and their constraining effects on the PP chains’ mobility [43]. For another, the larger specific surface area of the PA6 microfibrils provide less free volume for oxygen permeation. The two main factors could complicate the oxygen permeation path, and then improve the barrier properties of the PP/PA6 blends.

## 4. Conclusions

In this work, the evolution of the phase morphology and the crystallization behavior of the PP/PA6 polymer blends were studied systematically in the process of the “slit die extrusion-hot stretching”. By adjusting the velocity of the rollers, different stretching forces were applied on the PP/PA6 blends. As the stretching forces increased, the spherical PA6 domains evolved to be ellipsoid, rod-like and microfibrils, until the microfibrils broke up. In addition, the crystallization ability and crystal orientation degree of the PP matrix were also improved significantly in the PP/PA6 MFCs due to more excellent heterogeneous nucleation ability of the PA6 microfibrils. The results of DSC and POM showed that PP spherulite, fan-shaped lamellae and a transcrystalline layer formed respectively under the induction effects of the PA6 dispersed phases with different morphologies. In the PP/PA6 MFCs, the transcrystalline layers of the PP crystals showed a smaller average size, more crystals and stronger interface adhesion. Such internal structure significantly improved the elastic responses and oxygen barrier properties of the PP/PA6 polymer blends.

## Figures and Tables

**Table 1:** The thermal parameters for the non-isothermal crystallization.

Sample	*T*_0_(°C)	*T*_p_(°C)	*T*_m_(°C)	*X*_c_(%)
PP(*λ* = 1)	121.2	117.2	162.3	37.70
PP/PA6(*λ* = 1)	124.5	121.9	162.6	36.40
PP/PA6(*λ* = 2)	127.5	123.2	166.4	41.64
PP/PA6(*λ* = 7)	128.0	124.5	165.3	41.08
PP/PA6(*λ* = 11)	128.4	125.1	165.6	42.20
PP/PA6(*λ* = 16)	128.4	125.3	165.5	43.62

## Supplementary Materials

The following are available online at https://www.mdpi.com/2073-4360/12/4/878/s1. Table S1: Parameters of the pure PP and the PP/PA6 blends with different stretch ratios for the lattice planes (110) of the PP *α*-crystals from WAXS patterns. Table S2: Parameters of the pure PP and the PP/PA6 blends with different stretch ratios for the lattice planes (040) of the PP *α*-crystals from WAXS patterns.
