# Supplementary material for: The Formation of a Highly Oriented Structure and Improvement of Properties in PP/PA6 Polymer Blends during Extrusion-Stretching
Source: Polymers (Basel). 2020 Apr 10;12(4):878. doi: 10.3390/polym12040878 (PMC7240534; doi:10.3390/polym12040878)
Supplement: Supplementary file 1 [file polymers-12-00878-s001.pdf]

The Formation of a Highly Oriented Structure and Improvement of Properties in PP/PA6 Polymer Blends during Extrusion-Stretching

Yu Wang, Wenjie Sun, Song Liu, Huajian Ji, Xin Chen, Huihao Zhu, Haili Zhao, Yulu Ma, and Linsheng Xie*

Table S1. Parameters of the pure PP and the PP/PA6 blends with different stretch ratios for the lattice planes (110) of the PP α-crystals from WAXS patterns.

**Sample**	**2θ/°**	**β**	**1/βcos(θ)**
PP(λ = 1)	14.057	0.518	1.94512
PP/PA6(λ = 1)	14.057	0.603	1.67093
PP/PA6(λ = 2)	14.040	0.610	1.65172
PP/PA6(λ = 7)	14.080	0.614	1.64103
PP/PA6(λ = 11)	14.100	0.628	1.60449
PP/PA6(λ = 16)	14.080	0.635	1.58677

Table S2. Parameters of the pure PP and the PP/PA6 blends with different stretch ratios for the lattice planes (040) of the PP α-crystals from WAXS patterns.

**Sample**	**2θ/°**	**β**	**1/βcos(θ)**
PP(λ = 1)	16.846	0.455	2.22177
PP/PA6(λ = 1)	16.877	0.552	1.83142
PP/PA6(λ = 2)	16.820	0.553	1.82797
PP/PA6(λ = 7)	16.860	0.555	1.82148
PP/PA6(λ = 11)	16.880	0.566	1.78613
PP/PA6(λ = 16)	16.880	0.575	1.75817
